# Whole-genome resequencing reveals population genetic structure and selective sweeps in *Bombus waltoni*

**DOI:** 10.1186/s12864-026-13175-y

**Published:** 2026-07-16

**Authors:** Rui Zhang, Chengbo Liang, Wenrui Jiao, Lei Si, Daoxin Liu, Jingyan Yan

**Affiliations:** 1 College of Eco–Environmental Engineering, Qinghai University, Xining, 810016 China; 2 Qinghai Provincial Key Laboratory of Animal Ecological Genomics, Xining, 810016 China; 3 College of Agriculture and Animal Husbandry, Qinghai University, Xining, 810016 China

**Keywords:** Bumblebees, Genetic diversity, Population genetic structure, Selective sweeps, Qinghai–Tibetan Plateau

## Abstract

**Background:**

*Bombus waltoni* is a typical alpine bumblebee species and an important indicator species of the Qinghai-Tibetan Plateau. However, whole-genome studies of bumblebees from this region remain limited. In this study, we investigated the genetic diversity, population structure, and adaptive genomic variation of *B. waltoni*, providing essential genomic resources for understanding its population genetic structure and signatures of local adaptation.

**Results:**

Based on whole-genome SNP data, genetic diversity was relatively similar across *B. waltoni* populations, with expected heterozygosity (*He*) ranging from 0.2265 to 0.2601, unbiased expected heterozygosity (*uHe*) from 0.2517 to 0.2729, and nucleotide diversity (*π*) from 0.00104504 to 0.00113178. Genetic diversity was significantly associated with elevation after correction for multiple testing. Population structure analyses consistently resolved the 18 populations into two major genetic clusters corresponding to northeastern and southwestern regions, a pattern supported by ADMIXTURE, PCA, and phylogenetic analyses. Although overall genetic differentiation was low, a significant pattern of isolation by distance was detected. Multiple gene flow analyses suggested that population differentiation was inconsistent with complete isolation. Selective sweep analyses identified distinct sets of candidate genes and functional enrichment patterns between the two clusters.

**Conclusion:**

Significant but low genetic differentiation was detected among *B. waltoni* populations within the northeastern Qinghai–Tibetan Plateau, and populations showed relatively similar levels of genetic diversity. Gene flow signals suggested that population differentiation was inconsistent with complete isolation, while multiple genomic regions under selection suggest potential adaptive responses to elevation- and latitude-associated environmental heterogeneity. These results provide genomic evidence for population differentiation and local adaptation in a high-altitude pollinator, offering a scientific basis for region-specific conservation and management.

**Supplementary Information:**

The online version contains supplementary material available at https://doi.org/10.1186/s12864-026-13175-y.

## Introduction

The northeastern Qinghai-Tibet Plateau is characterized by a cold climate, with vegetation dominated by alpine meadows [1]. This distinctive environment has made bumblebees one of the primary pollinator groups in the region. Bumblebees possess long proboscises and strong foraging abilities, along with high pollination efficiency, cold tolerance, and strong flight capacity [2]. Through thermoregulation, they are able to maintain internal physiological stability, enabling them to cope with extreme environmental conditions associated with high altitude, low temperature, and large diurnal temperature fluctuations. The transition zone from the eastern Qinghai–Tibet Plateau to the Loess Plateau, Qinling Mountains, and Sichuan Basin is one of the global hotspots of bumblebee diversity [3]. *B. waltoni* belongs to the subgenus *Mendacibombus* [4] and is among the key indicator species of the Qinghai-Tibet Plateau [5]. It is primarily distributed across the plateau and its eastern Himalayan–Hengduan mountain margin and represents a typical alpine bumblebee [6]. In this complex and variable environment, *B. waltoni* has likely undergone substantial genetic differentiation and environmental adaptation. Moreover, pollinator populations worldwide have declined significantly in recent years due to the combined effects of climate change, pathogen infection, and habitat loss [7, 8]. Investigating the genetic characteristics and environmental adaptations of *B. waltoni* provides insights into pollinators' adaptive evolution on the plateau and offers critical scientific evidence for maintaining ecosystem stability and pollination services in this high-altitude region.

In recent years, whole-genome–based studies of the genus *Bombus* have achieved significant progress. Sun et al. [9] sequenced and assembled 17 representative species across all 15 subgenera, providing comprehensive insights into genetic and genomic variation at the genus level. Their study revealed stable chromosomal karyotypes and dynamically evolving gene families, and identified signals of positive selection associated with key traits, such as foraging behavior and adaptation to high-altitude environments. These findings established a solid genomic foundation for understanding the adaptive evolution of bumblebees. Advances in whole-genome resequencing technologies have also enabled the investigation of species’ adaptive responses to environmental heterogeneity at the population level. Accumulating evidence indicates that different bumblebee species exhibit distinct evolutionary strategies in complex environments. For example, *B. vosnesenskii*, a widely distributed species in western North America, showed weak population genetic structure and limited signals of environment-associated local adaptation [10], reflecting its broad environmental flexibility. In contrast, landscape genomics analyses of *B. vancouverensis*, a species inhabiting the mountainous regions of western North America, revealed pronounced local adaptation [11]. Isothermality and elevation were identified as key environmental drivers of genomic differentiation, and multiple candidate genes related to temperature tolerance and hypoxia response were associated with high-altitude adaptation. These studies demonstrate the power of whole-genome population genomic approaches in elucidating adaptive evolution and emphasize the decisive role of species’ ecological traits and habitat heterogeneity in shaping genomic variation patterns.

However, whole-genome population genomic studies of bumblebees from the Qinghai-Tibet Plateau remain limited, particularly with respect to systematic analyses of their genetic characteristics and adaptive evolutionary processes in high-altitude environments. To address this gap, we used whole-genome resequencing data to systematically examine populations of *B. waltoni* from the northeastern Qinghai-Tibet Plateau. Specifically, we assessed genetic diversity and its associations with geographic and environmental variables, characterized population genetic structure, differentiation patterns, and gene flow, and further explored the molecular basis of potential adaptive evolution as reflected by signals of selective sweeps in alpine environments.

## Materials and methods

### Sample collection and data acquisition

**Fig. 1:**
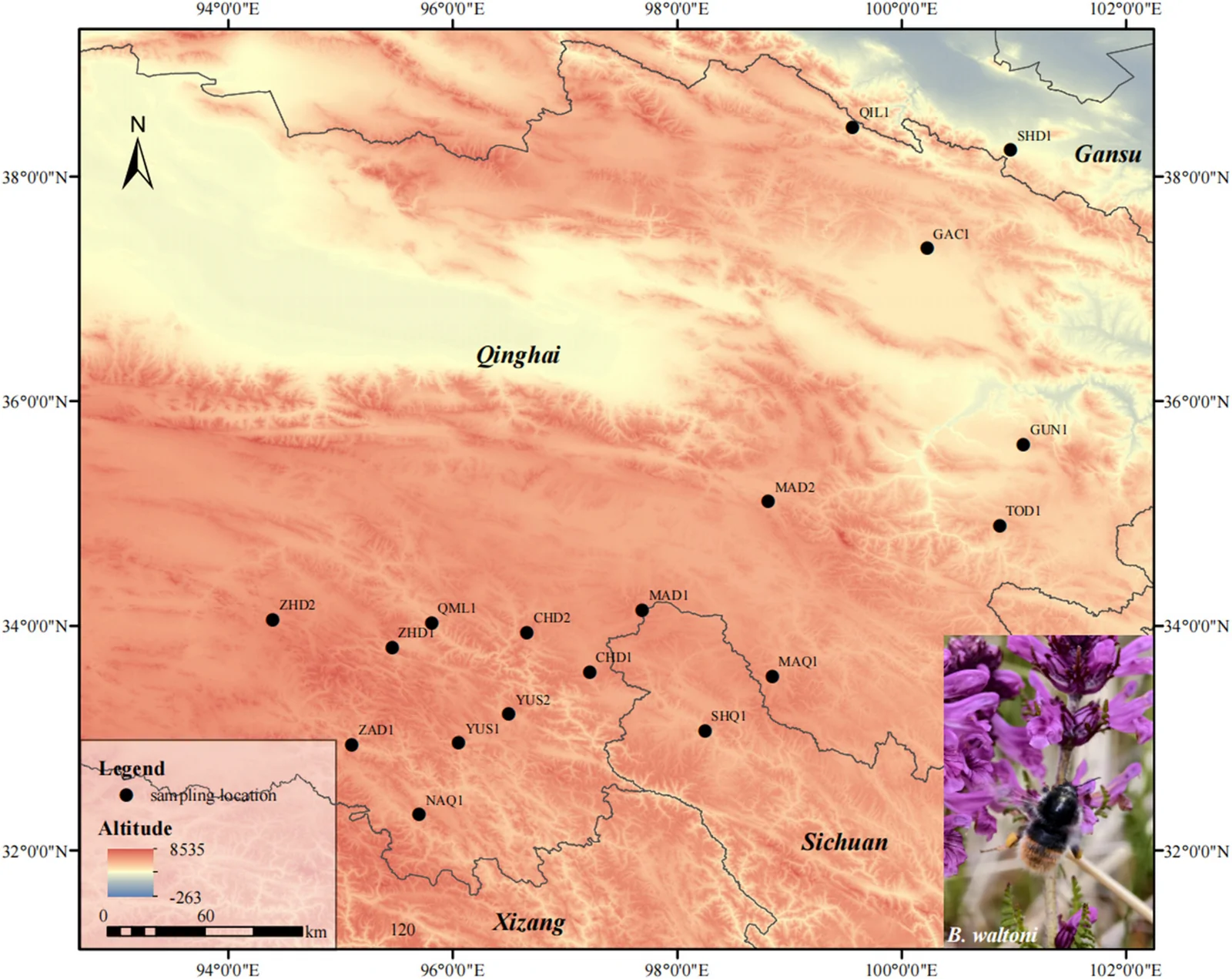
Geographic locations of the *B. waltoni* sampling sites

*B. waltoni* workers were collected from the northeastern Qinghai-Tibet Plateau using sweep netting. At each sampling site, the sampling area was defined as approximately 1 km², and collections were conducted for 1 h. Location coordinates and site-specific information were recorded and labeled accordingly (Fig. 1, Table S1). A total of 146 individuals were collected from 18 sampling sites, with 5 to 14 individuals captured per site. Following species identification, thoracic muscle tissue was collected from each individual and sent to Shanghai Majorbio Bio-Pharm Technology Co., Ltd. (Shanghai, China) for DNA library preparation and whole-genome resequencing. Qualified libraries were sequenced on the Illumina NovaSeq platform using a paired-end 150 bp (PE150) strategy, yielding a total read length of 300 bp per fragment.

### Sequencing data processing

Raw sequencing reads were subjected to quality control using fastp v0.24.1 [12], including automatic adapter detection and removal, trimming of low-quality bases at both read ends, removal of poly-G tails, and filtering of reads with an average end-base quality score < 20, more than 10 unknown bases (N), or a length < 25 bp. Sequencing data statistics before and after quality control were summarized using custom Python scripts. High-quality reads passing these filters were aligned to the *B. waltoni* reference genome (NCBI assembly GCA_014737395.1) using the MEM algorithm of BWA v0.7.17 [13]. The resulting alignments were sorted using SAMtools v1.5 [14], and PCR duplicates were removed with the MarkDuplicates module in GATK v4.1.8.1 [15]. Sequencing depth and genome coverage for each sample were calculated using SAMtools and custom Python scripts. Variant calling was performed using the GATK workflow. Briefly, the HaplotypeCaller module was applied to each sample to generate gVCF files, which were subsequently subjected to joint genotyping using CombineGVCFs and GenotypeGVCFs. SNP sites were extracted using SelectVariants and further filtered using VariantFiltration with the following criteria: Quality by Depth (QD) < 2.0, Mapping Quality (MQ) < 40.0, Fisher Strand bias (FS) > 60.0, Strand Odds Ratio (SOR) > 3.0, Mapping Quality Rank Sum (MQRankSum) < − 12.5, and Read Position Rank Sum (ReadPosRankSum) < − 8.0. High-quality SNPs were functionally annotated using SnpEff v5.2e [16].

Because all samples were collected from wild populations and their relatedness could not be determined a priori, pairwise PI_HAT values were calculated using the --genome option in PLINK v1.9.0 [17] to evaluate identity by descent among individuals. PI_HAT represents the estimated proportion of the genome shared identical-by-descent. According to Chen et al. [18], PI_HAT values of 0, 0.25, 0.5, and 1 correspond to unrelated individuals, first-cousin-level relatedness, parent–offspring or full-sibling relationships, and the same individual or monozygotic twins, respectively. Therefore, sample pairs with PI_HAT > 0.25 were treated as putatively close relatives, and only one representative individual from each related pair was retained for subsequent analyses. As a result, 139 mutually independent individuals were obtained for subsequent analyses (Table S2, Fig. S1). After GATK hard filtering, a total of 5,269,939 SNPs were retained across the 139 individuals.

### Genetic diversity of *B. waltoni* populations and its influencing factors

SNP filtering was conducted in PLINK v1.9 using the following parameters: --geno 0.05, --maf 0.05, --hwe 1e − 6, and --biallelic-only. After filtering, 980,746 high-quality SNPs were retained. Observed heterozygosity (*Ho*) was calculated using the --het option in PLINK, while allele frequencies were obtained using the --freq option. Expected heterozygosity (*He*), unbiased expected heterozygosity (*uHe*; expected heterozygosity corrected for finite sample size), inbreeding coefficient (*Fis*), polymorphism information content (*PIC*; an index reflecting the informativeness and polymorphism level of SNP loci based on allele frequencies), and Shannon’s diversity index (*I*) were subsequently calculated at the population level based on allele frequency data. In addition, nucleotide diversity (*π*) was estimated using VCFtools v0.1.16 [19] with a sliding window size of 10 kb and a step size of 5 kb. All genetic diversity indices were summarized at the population level and used to compare genetic diversity among populations.

To further investigate the factors influencing genetic diversity in *B. waltoni* populations, geographic parameters and environmental variables were extracted for each population using the *terra* package (v1.8.80) in R v4.4.1. To improve interpretability and reduce redundancy among diversity measures, we focused on four genetic diversity indices representing different aspects of population genetic variation: *Ho*, *uHe*, *Fis*, and *π*. These indices reflect observed heterozygosity, expected gene diversity corrected for sample size, within-population heterozygote deficit or excess, and sequence-level nucleotide variation, respectively. Candidate predictors included three geographic parameters (longitude, latitude, and elevation) and 26 environmental variables, including annual precipitation, annual mean temperature, inter-patch dissimilarity, herbaceous vegetation cover, topographic position index, aspect, and monthly precipitation, temperature, solar radiation, wind speed, and water vapor pressure from June to September. Climatic variables were obtained from the WorldClim global climate database (https://www.worldclim.org), representing long-term climatic averages for the period 1970–2000, while land cover, habitat heterogeneity, and topographic variables were derived from the EarthEnv database (https://www.earthenv.org). All environmental variables were extracted at an approximate spatial resolution of 1 km². To reduce multicollinearity among predictors, pairwise Pearson correlation coefficients were calculated among all candidate predictors, and one variable from each highly correlated pair (|r| > 0.8) was removed based on ecological relevance and interpretability. After filtering, longitude, elevation, topographic position index, annual precipitation, annual mean temperature, inter-patch dissimilarity, herbaceous vegetation cover, aspect, and July wind speed were retained for downstream analyses (Fig. S2). Pearson correlation analyses were then conducted between these retained variables and the four genetic diversity indices, and *P* values were adjusted using the Benjamini–Hochberg false discovery rate (FDR) procedure. Correlation matrices were visualized using the *corrplot* package (v0.95) and *ggplot2* package (v3.5.1) in R.

### Population genetic structure analyses

Population genetic structure was investigated using the filtered high-quality SNP dataset. To minimize the effect of linkage disequilibrium (LD), SNP pruning was performed in PLINK using the --indep-pairwise 50 5 0.2 option (window size = 50 SNPs, step size = 5 SNPs, r² threshold = 0.2). After LD pruning, 704,528 SNPs were retained and used for subsequent population structure analyses. Ancestry inference was conducted using ADMIXTURE v1.3.0 [20], with the number of ancestral populations (K) ranging from 1 to 10. For each K value, cross-validation (CV) errors were calculated, and the optimal K was determined based on the minimum CV error. Principal component analysis (PCA) was performed in PLINK using the LD-pruned SNP dataset, and the first 10 principal components were extracted. The first two principal components were used to visualize genetic clustering patterns among individuals. To infer phylogenetic relationships, one individual of *B. rufofasciatus*, which belongs to the subgenus *Melanobombus*, was included as an outgroup. This *B. rufofasciatus* individual was sequenced in this study, and its sequencing data have been deposited in the National Genomics Data Center (NGDC) under accession number CRA042239. Sequencing reads from both *B. waltoni* and the outgroup were aligned to the *B. waltoni* reference genome to ensure consistent SNP coordinates. Genetic distances among 139 *B. waltoni* individuals and the outgroup were calculated using PLINK, and a neighbor-joining (NJ) tree was constructed based on the resulting distance matrix using PHYLIP [21], with 1,000 bootstrap replicates to assess node support. Additionally, molecular variance analysis (AMOVA) was performed using the *poppr* package (v2.9.8) in R, based on the population clusters inferred from ADMIXTURE at the optimal K value, to evaluate genetic differentiation among groups. Statistical significance was assessed using 9,999 permutation tests. Pairwise genetic differentiation among populations was estimated using VCFtools. The degree of genetic differentiation was interpreted according to Wright’s criteria [22]: *F*st < 0.05 indicates low genetic differentiation, 0.05 < *F*st < 0.15 indicates moderate genetic differentiation, and 0.15 < *F*st < 0.25 indicates high genetic differentiation. Gene flow among populations was approximately estimated from pairwise *F*st values using the formula *N*m = (1 − *F*st) / 4*F*st. Finally, pairwise *F*st and *N*m matrices were visualized as heatmaps in R using the *ggplot2* package.

### Gene flow analyses

To detect potential gene flow among populations, *D*-statistics (ABBA–BABA tests) were performed using Dsuite [23], with *B. rufofasciatus* designated as the outgroup. The D-statistic measures the normalized difference between ABBA and BABA site patterns in a four-taxon framework (P1, P2, P3, and an outgroup). Significant deviations from zero indicate asymmetric sharing of derived alleles, which may suggest introgression or gene flow beyond the expectation under incomplete lineage sorting alone. Analyses were conducted using the LD-pruned dataset consisting of 704,528 SNPs to minimize the influence of linkage disequilibrium. The Dtrios module was used to calculate *D*-statistics and f4-ratios. Statistical significance was assessed using Z-scores, and tests with |Z| ≥ 3 were considered significant. The F-branch module in Dsuite was applied to estimate excess allele sharing along specific branches of the inferred phylogeny using the same outgroup (*B. rufofasciatus*). The *fb* value represents the estimated proportion of ancestry or allele sharing attributable to a specific branch based on the f4-ratio statistic. Higher *fb* values indicate stronger excess allele sharing between the corresponding branch and another population or branch, suggesting stronger potential introgression or gene flow signals, whereas lower *fb* values indicate weaker or no detectable excess allele sharing [23]. Results were visualized using the dtools.py script. To further infer historical gene flow among populations, TreeMix [24] was employed based on the same LD-pruned SNP dataset. In the TreeMix analysis, no outgroup was specified, and migration events were modeled under an unrooted framework. The number of migration events (m) was set from 0 to 10, and 50 independent replicates were performed for each m value to ensure convergence. The optimal number of migration events was determined using the *OptM* package (v0.1.9) in R. Final models were visualized using the plotting_funcs.R script.

### Selective sweep analysis

Selective sweep analysis was performed to identify genomic regions potentially associated with adaptive variation [25]. Based on the filtered high-quality SNP dataset consisting of 980,746 SNPs, nucleotide diversity (*π*) was calculated separately for the two genetic clusters identified by population structure analyses, and genetic differentiation (*F*st) between the two groups was estimated using VCFtools. Selective sweep signals were detected using a sliding-window approach with a window size of 10 kb and a step size of 5 kb. Windows containing fewer than five SNPs were excluded to reduce false-positive signals. Genomic windows with *F*st values in the top 5% of the whole-genome distribution were considered highly differentiated regions and were further filtered based on the distribution of log₂(θπ_southwest_ / θπ_northeast_). Specifically, windows with *F*st values in the top 5% and log₂(θπ_southwest_ / θπ_northeast_) values in the upper 5% were defined as candidate selective sweep regions in the northeastern populations, whereas windows with *F*st values in the top 5% and log₂(θπ_southwest_ / θπ_northeast_) values in the lower 5% were defined as candidate selective sweep regions in the southwestern populations. Genes located within candidate selective sweep regions were considered putative targets of selection. The available *B. waltoni* protein sequences retrieved from NCBI were functionally annotated using the eggNOG-mapper web server, and the resulting Gene Ontology (GO) terms and Kyoto Encyclopedia of Genes and Genomes (KEGG) pathway annotations were used for downstream enrichment analyses of candidate genes. GO and KEGG enrichment analyses were conducted in TBtools v2.422, and the results were visualized using the *ggplot2* package in R.

## Results

### Whole-genome resequencing data quality

Whole-genome resequencing was performed for 146 *B. waltoni* individuals, generating approximately 515.13 Gb of raw sequencing data, with an average of 23,366,299 raw reads per sample. After quality control, a total of 501.50 Gb of high-quality clean data was obtained, with an average of 23,130,500 clean reads per sample. The average proportion of effective data was 97.37%, and the average GC content was 40.81%. The average Q20 and Q30 values were 97.39% and 94.75%, respectively (Table S3), indicating that the sequencing data were of high overall quality. After mapping the clean reads to the *B. waltoni* reference genome, the mapping rate ranged from 64.91% to 99.57% across samples, with an average mapping rate of 97.77% (Table S4). The average sequencing depth was 12.59×. In addition, an average of 99.62% of the reference genome was covered at a depth of ≥ 1×, and 97.40% was covered at a depth of ≥ 4×, indicating high sequencing coverage and sufficient data quality and genome coverage for subsequent variant detection.

### Genetic diversity analysis of *B. waltoni* populations

Genetic diversity was assessed across *B. waltoni* populations based on whole-genome SNP data. Overall, diversity indices were broadly similar among populations (Table 1). *Ho* ranged from 0.2792 to 0.3481 (mean = 0.3130), whereas *He* ranged from 0.2265 to 0.2601 (mean = 0.2491). *uHe* varied from 0.2517 to 0.2729 (mean = 0.2678). Across all populations, *Ho* consistently exceeded *He*, and *Fis* values were negative (− 0.4395 to − 0.0921), indicating heterozygote excess. *PIC* ranged from 0.1837 to 0.2141 (mean = 0.2039), while *I* varied from 0.4973 to 0.5868 (mean = 0.5556). *π* ranged from 0.00104504 to 0.00113178 (mean = 0.00110945). Although overall diversity levels were comparable among populations, the GAC1, QIL1, and SHD1 populations exhibited relatively lower values across multiple diversity indices.

**Table 1:** Genetic diversity of different *B. waltoni* populations

POP	Ho	He	uHe	PIC	I	Fis	π
NAQ1	0.3481	0.2435	0.2706	0.1984	0.5377	-0.4294	0.00112413
ZAD1	0.2840	0.2601	0.2714	0.2141	0.5868	-0.0921	0.00112155
ZHD1	0.3014	0.2574	0.2726	0.2114	0.5778	-0.1707	0.00112926
ZHD2	0.3000	0.2562	0.2712	0.2103	0.5747	-0.1713	0.00112377
YUS1	0.2967	0.2563	0.2714	0.2106	0.5755	-0.1576	0.00112328
YUS2	0.3267	0.2483	0.2709	0.2029	0.5517	-0.3159	0.00112368
QML1	0.3007	0.2534	0.2703	0.2080	0.5677	-0.1864	0.00111730
CHD1	0.3182	0.2481	0.2707	0.2027	0.5508	-0.2826	0.00112307
CHD2	0.3262	0.2479	0.2705	0.2026	0.5507	-0.3158	0.00112181
SHQ1	0.3430	0.2423	0.2693	0.1975	0.5352	-0.4154	0.00111738
MAD1	0.2945	0.2568	0.2719	0.2110	0.5768	-0.1470	0.00112425
MAD2	0.3309	0.2502	0.2729	0.2044	0.5557	-0.3226	0.00113178
MAQ1	0.3038	0.2549	0.2719	0.2092	0.5710	-0.1919	0.00112559
TOD1	0.2953	0.2550	0.2701	0.2096	0.5730	-0.1580	0.00111671
GUN1	0.3055	0.2518	0.2686	0.2064	0.5632	-0.2133	0.00110617
GAC1	0.3472	0.2265	0.2517	0.1837	0.4973	-0.5328	0.00104764
QIL1	0.3325	0.2310	0.2520	0.1879	0.5099	-0.4395	0.00104772
SHD1	0.2792	0.2430	0.2528	0.1991	0.5452	-0.1489	0.00104504

*POP* population code, *Ho* observed heterozygosity, *He* expected heterozygosity, *uHe* unbiased expected heterozygosity corrected for finite sample size, *PIC* polymorphism information content, an index reflecting the informativeness and polymorphism level of SNP loci based on allele frequencies, *I* Shannon’s diversity index, *Fis* inbreeding coefficient, reflecting the relative deficit or excess of heterozygotes within populations, *π* nucleotide diversity

### Correlation between genetic diversity and environmental factors in *B. waltoni* populations

**Fig. 2:**
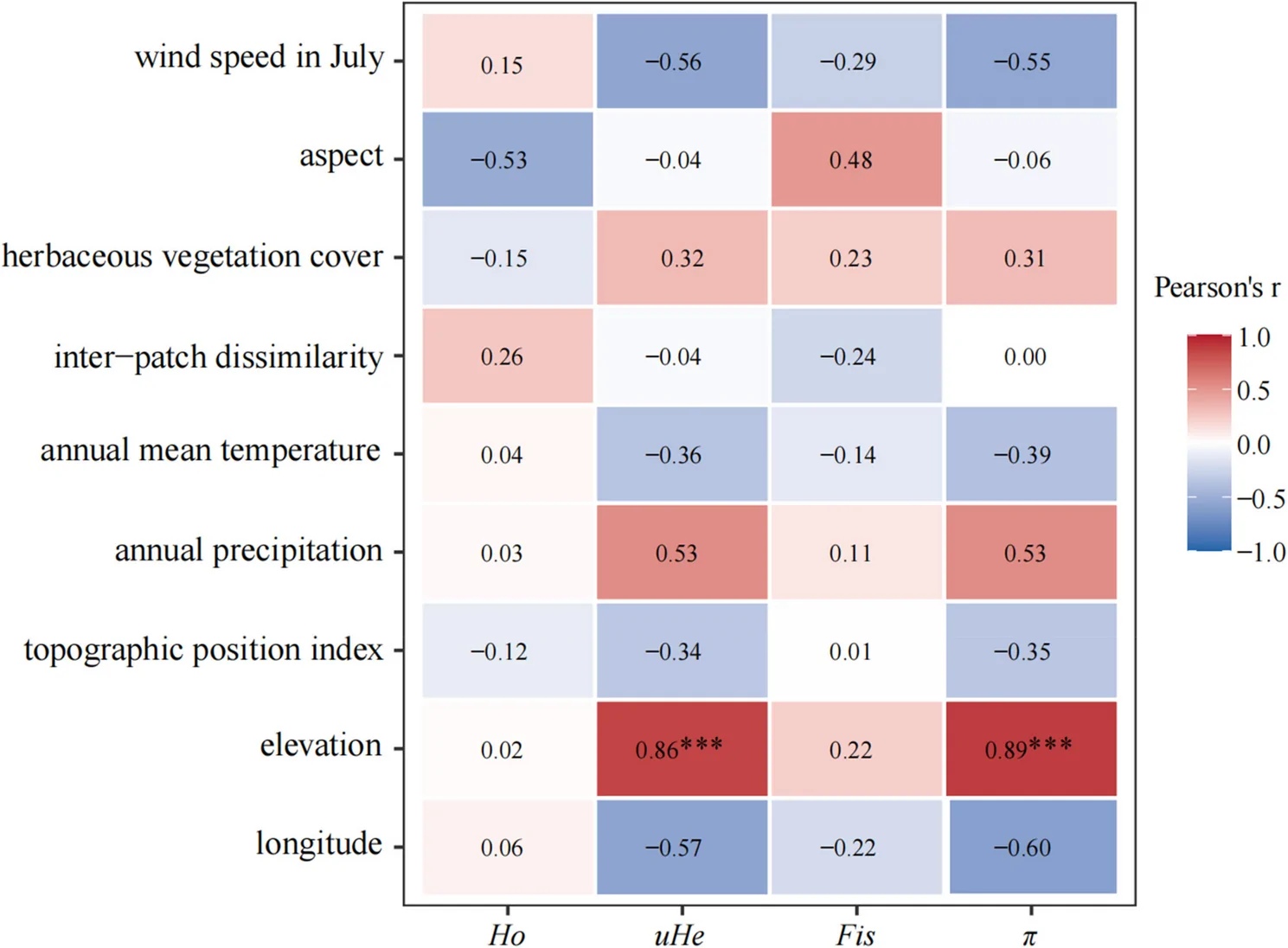
Correlations between population genetic diversity and environmental factors. *Ho*: observed heterozygosity; *uHe*: unbiased expected heterozygosity; *Fis*: inbreeding coefficient; *π*: nucleotide diversity; Asterisks indicate significance after Benjamini–Hochberg FDR correction (* FDR-adjusted *P* < 0.05, ** FDR-adjusted *P* < 0.01, *** FDR-adjusted *P* < 0.001)

Pearson correlation analyses were conducted to assess the relationships between four genetic diversity indices (*Ho*, *uHe*, *Fis*, and *π*) and nine environmental variables retained after collinearity filtering (Fig. 2). Correlation patterns varied among genetic diversity indices. Overall, *uHe* and *π* showed similar trends, being negatively associated with longitude, topographic position index, annual mean temperature, and July wind speed, and positively associated with elevation, annual precipitation, and herbaceous vegetation cover. In contrast, *Ho* and *Fis* showed generally weaker associations with environmental variables. After FDR correction, elevation was the only environmental factor significantly associated with genetic diversity, with both *uHe* (*r* = 0.86) and *π* (*r* = 0.89) showing significant positive correlations with elevation (FDR-adjusted *P* < 0.001). Although the remaining environmental variables showed varying degrees of association with different genetic diversity indices, none remained significant after multiple-testing correction.

### Analysis of population genetic structure and differentiation in *B. waltoni*

**Fig. 3:**
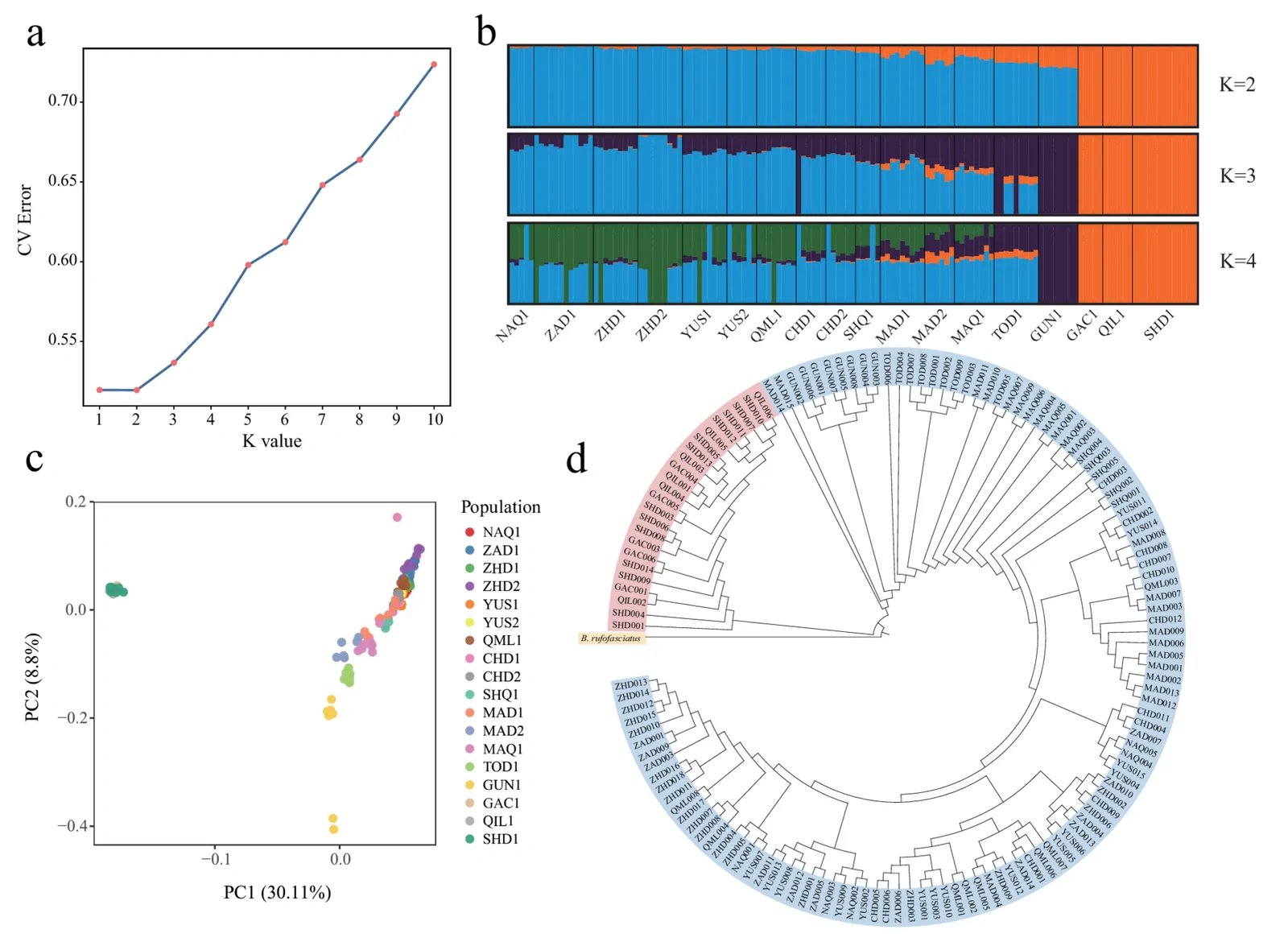
Population genetic structure and differentiation of *B. waltoni*. **a** CV error values from ADMIXTURE analyses for K values ranging from 1 to 10; **b** ADMIXTURE clustering results at K = 2, 3, and 4; **c** PCA based on whole-genome SNPs; **d** NJ phylogenetic tree constructed from whole-genome SNP data

Ancestry inference was conducted for 18 *B. waltoni* populations using ADMIXTURE, with K values ranging from 1 to 10. The cross-validation (CV) error reached its minimum at K = 2 (Fig. 3a), supporting the presence of two major genetic clusters. At K = 2–4 (Fig. 3b), the northeastern populations (GAC1, QIL1, and SHD1) consistently formed one cluster, whereas the remaining southwestern populations grouped into the other cluster. Across increasing K values, the northeastern populations remained genetically distinct. PCA revealed a similar pattern (Fig. 3c). PC1 explained 30.11% of the total genetic variation and clearly separated GAC1, QIL1, and SHD1 from the other populations. Phylogenetic analysis based on whole-genome SNP data further resolved the populations into two major clades (Fig. 3d), corresponding to the northeastern and southwestern clusters. The congruence among ADMIXTURE, PCA, and phylogenetic analyses consistently supported a two-cluster population structure in *B. waltoni*.

**Fig. 4:**
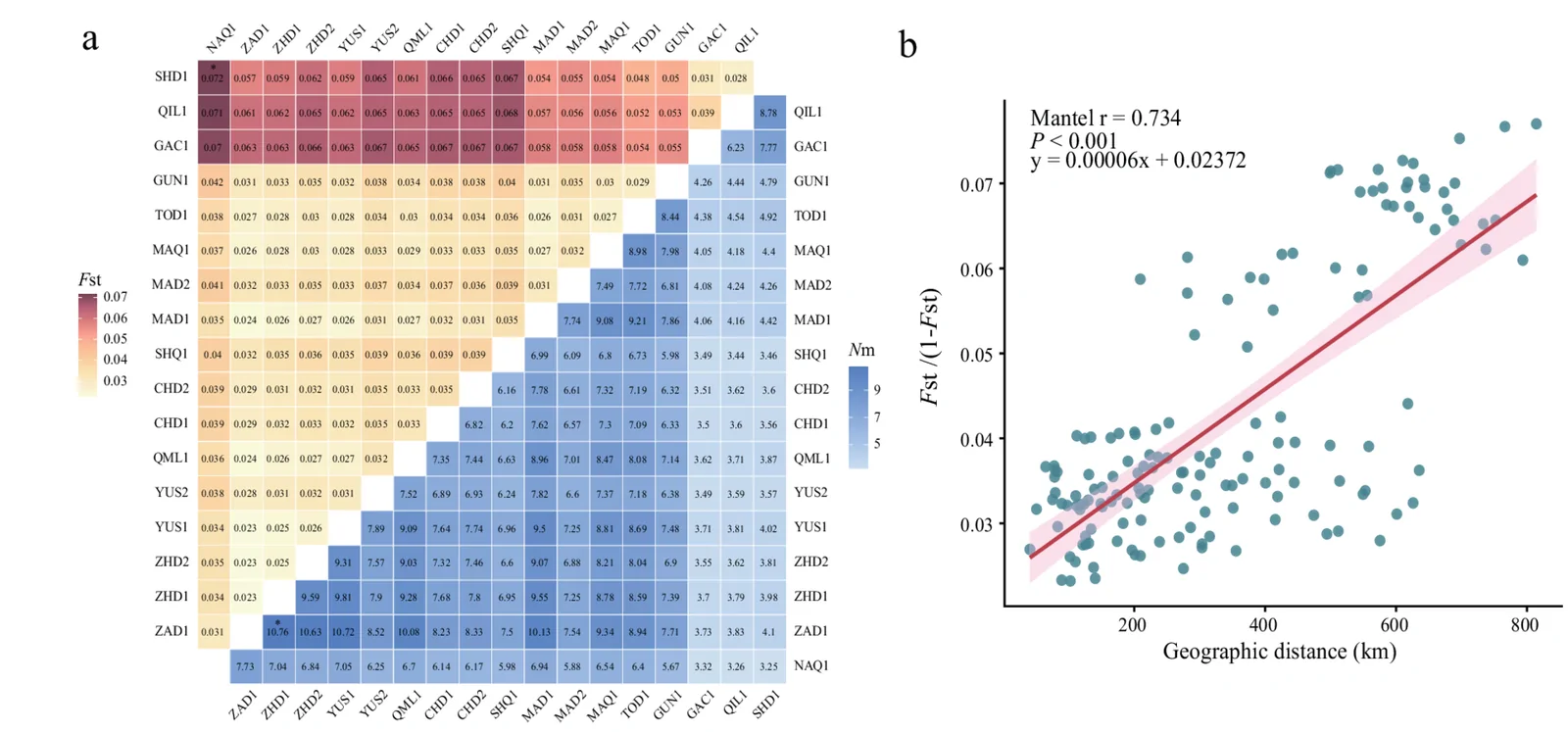
Genetic differentiation and gene flow among *B. waltoni* populations and their relationship with geographic distance. **a** Pairwise matrix of genetic differentiation (*F*st; upper triangle) and gene flow (*N*m; lower triangle); **b** Mantel test showing the correlation between genetic differentiation and geographic distance

Genetic differentiation and gene flow among *B. waltoni* populations were further assessed. Pairwise *F*st values ranged from 0.023 to 0.072, with a mean of 0.041 (Fig. 4a), reflecting generally low levels of genetic differentiation. Correspondingly, estimates of gene flow (*N*m) ranged from 3.25 to 10.76, with an average of 6.55. The highest genetic differentiation and lowest gene flow were observed between the NAQ1 and SHD1 populations (Tables S5 and S6). In contrast, the ZAD1 and ZHD1 populations exhibited the lowest genetic differentiation (*F*st = 0.022697) and the highest gene flow. Consistent with the population structure analyses, low to moderate genetic differentiation was detected between the northeastern and southwestern genetic clusters (*F*st = 0.048–0.072). Mantel test analysis revealed a significant positive correlation between genetic differentiation and geographic distance (*r* = 0.734, *P* < 0.001; Fig. 4b), indicating a clear pattern of isolation by distance.

### Molecular variance analysis (AMOVA) of *B. waltoni* populations

AMOVA was conducted based on the two genetic clusters identified by ADMIXTURE (K = 2) to partition genetic variation across hierarchical levels. The analysis revealed significant genetic differentiation among groups (*P* = 0.0001; Table 2). Most of the total genetic variation was distributed within individuals (89.85%). The remaining variation was attributed to differences among groups (5.04%), among populations within groups (0.33%), and among individuals within populations (4.78%). Although the proportion of variation among groups was modest, it exceeded that observed among populations within groups, consistent with genetic differentiation between the northeastern and southwestern clusters.

**Table 2:** AMOVA of population genetic structure in *B. waltoni* at K = 2

Source of Variation	df	Variance Components	Percentage of Variation	Fixation Indices	*P*-value
among groups	1	7017.52 Va	5.04	FCT = 0.0504	0.0001
among populations within groups	16	461.84 Vb	0.33	FSC = 0.0035	0.0001
among individuals within populations	121	6654.78 Vc	4.78	FIS = 0.0505	0.0005
within individuals	139	125069.34 Vd	89.85	/	0.0001

### Gene flow analysis among *B. waltoni* populations

**Fig. 5:**
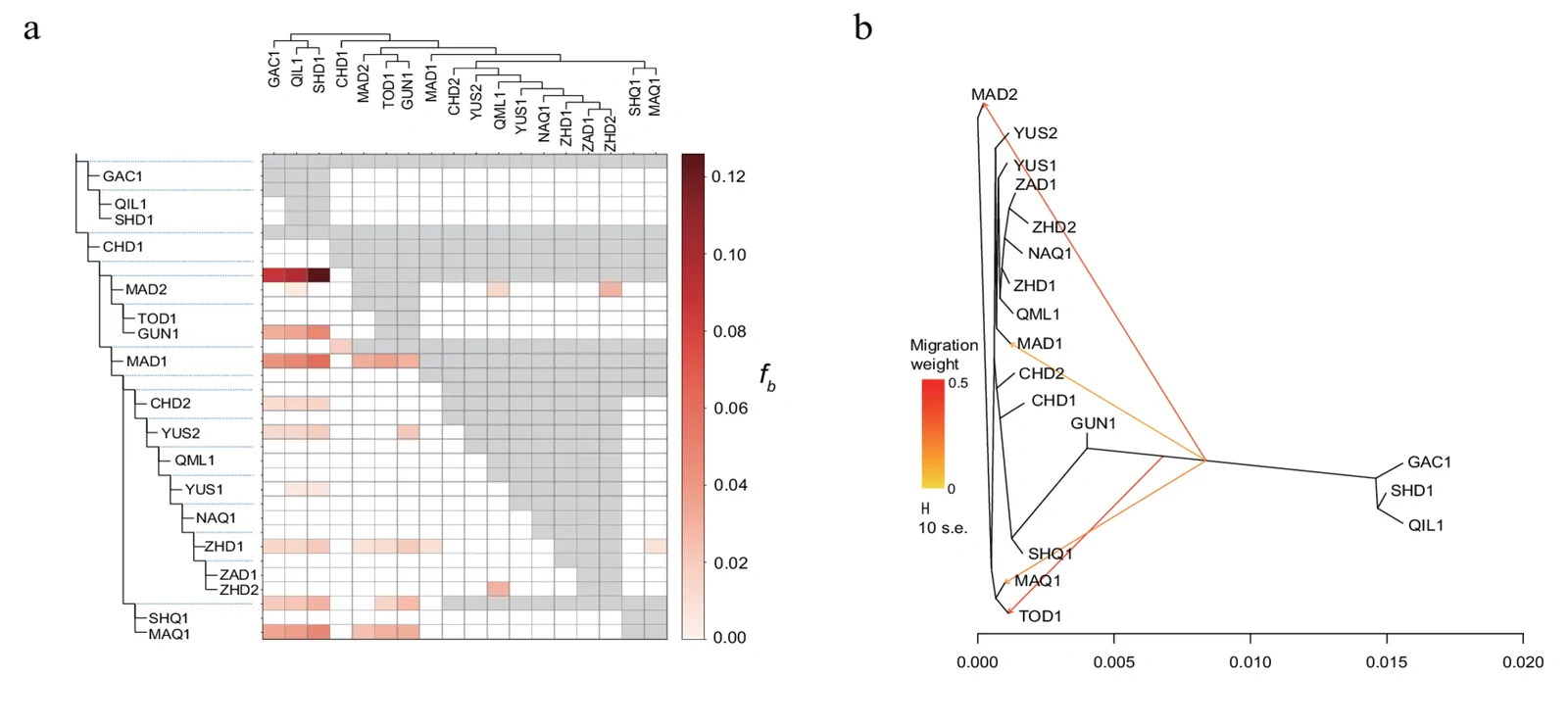
F-branch and TreeMix inference of historical gene flow among populations. **a ***f*_*b*_ values from F-branch analysis based on the Dsuite framework with *B. rufofasciatus* as the outgroup; **b** TreeMix population graph with four migration events (m = 4) inferred without specifying an outgroup

ABBA–BABA tests implemented in Dtrios were conducted to evaluate excess allele sharing among populations. Among 816 tested population trios, 321 exhibited significant D-statistics (|Z| ≥ 3, *P* < 0.01; Table S7), consistent with asymmetric allele sharing among subsets of populations. F-branch analyses based on the f4-ratio statistic revealed elevated *f*_*b*_ values, indicating stronger excess allele sharing between several southwestern population branches and the northeastern populations (GAC1, QIL1, and SHD1) (Fig. 5a; Table S8). In particular, branches containing MAD2, TOD1, and GUN1 showed the highest *f*_*b*_ values relative to the northeastern population. TreeMix analysis further explored historical relationships among populations. Under the model without migration (m = 0), populations clustered primarily according to genetic similarity (Fig. S3), with GAC1, QIL1, and SHD1 forming a distinct lineage. As the number of migration events increased, additional migration edges were inferred connecting the northeastern lineage to several southwestern populations. OptM analysis identified m = 4 as the optimal number of migration events (Fig. S4). Under this model, migration edges were inferred from the northeastern lineage to MAD2, MAD1, TOD1, and MAQ1 populations (Fig. 5b). Collectively, results from the ABBA–BABA tests, F-branch analysis, and TreeMix modeling support the presence of historical gene flow among *B. waltoni* populations.

### Selective sweep signals and functional enrichment

**Fig. 6:**
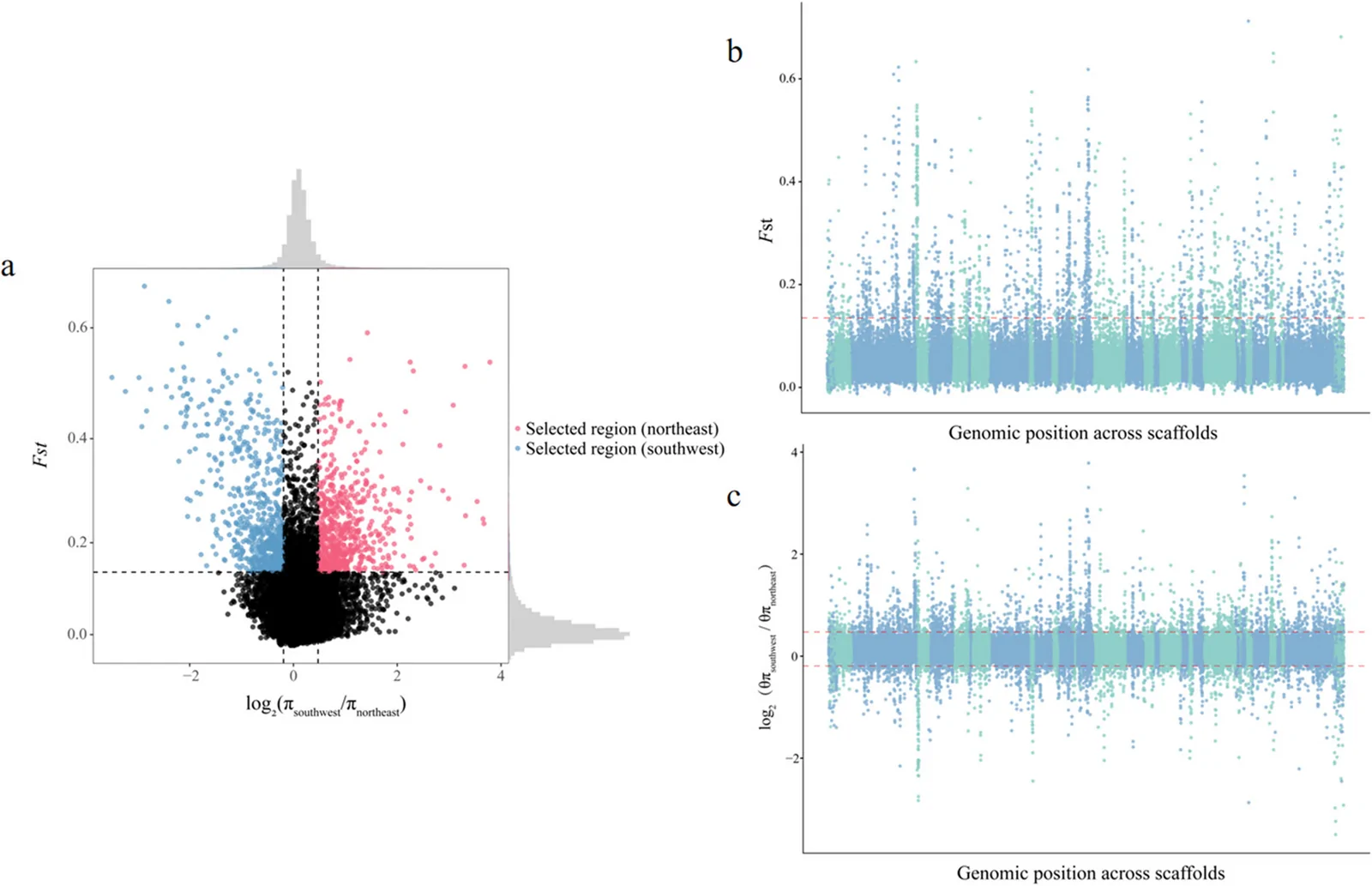
Whole-genome directional selection scans based on *F*st and π ratios. **a** Genomic regions showing strong signals of directional selection; **b** Whole-genome sliding-window scan of *F*st, with the dashed line indicating the top 5% threshold of the Whole-genome distribution; **c** Whole-genome sliding-window scan of log_2_(θ*π*_southwest_ / θ*π*_northeast_), with dashed lines indicating the upper and lower 5% thresholds. In panels b and c, the x-axis shows genomic positions across ordered scaffolds, and alternating colors indicate adjacent scaffolds. Individual scaffold labels are omitted to avoid overlap

**Fig. 7:**
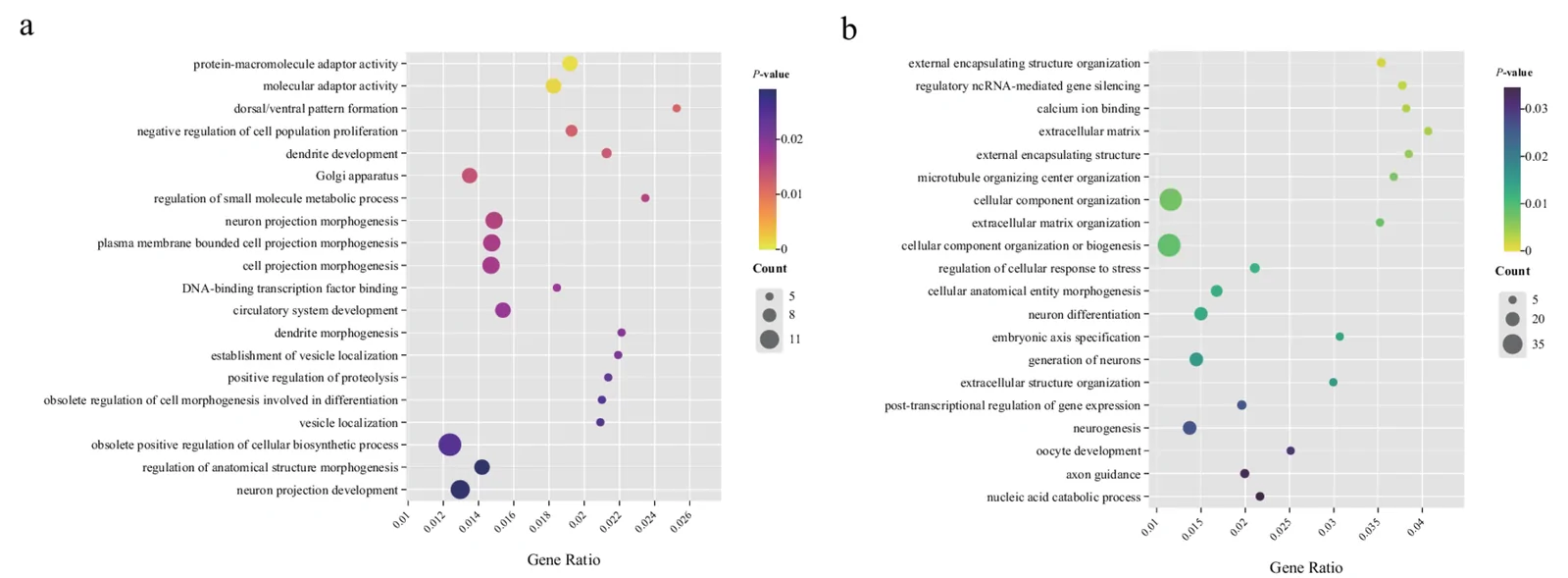
GO enrichment of genes under selection in the northeastern and southwestern cluster. **a** Northeastern cluster; **b** Southwestern cluster

Selective sweep signals were identified based on a combined analysis of *Fst* and log_2_(θ*π*_southwest_ / θ*π*_northeast_). A total of 10,814 candidate SNPs were detected in the northeastern cluster, corresponding to 78 candidate genes, whereas 9,966 candidate SNPs corresponding to 105 genes were identified in the southwestern cluster (Fig. 6). Functional enrichment analysis revealed 31 significantly enriched GO terms (Table S9) and five KEGG pathways (Table 3) in the northeastern cluster. In the southwestern cluster, 24 GO terms (Table S10) and two KEGG pathways (Table 4) were significantly enriched. The top 20 GO terms ranked by adjusted *P* value were further examined. In the northeastern cluster, candidate genes were enriched in GO categories associated with developmental processes, neural structure formation, cell morphogenesis, and intracellular transport and metabolic regulation (Fig. 7a). In contrast, candidate genes in the southwestern cluster were enriched in GO terms related to cellular component organization, regulation of gene expression, and stress response processes (Fig. 7b).

**Table 3:** KEGG pathway enrichment analysis of genes under selection in the northeastern cluster

KEGG Term	Main Class	Selected Set	Background	*P*-value
Other glycan degradation	Metabolism	2	17	0.0047
Terpenoid backbone biosynthesis	Metabolism	2	31	0.0151
SNARE interactions in vesicular transport	Genetic Information Processing	2	37	0.0212
Metabolism of terpenoids and polyketides	Metabolism	2	49	0.0357
Membrane trafficking	Brite Hierarchies	9	800	0.0481

**Table 4:** KEGG pathway enrichment analysis of genes under selection in the southwestern cluster

KEGG Term	Main Class	Selected Set	Background	*P*-value
Caffeine metabolism	Metabolism	1	2	0.0159
Type II diabetes mellitus	Human Diseases	2	28	0.0207

KEGG pathway analysis showed that genes under selection in the northeastern cluster were enriched in pathways related to membrane transport, vesicular trafficking, and secondary metabolite metabolism. In comparison, candidate genes in the southwestern cluster were enriched in pathways primarily associated with energy metabolism and xenobiotic metabolism. Overall, the functional enrichment patterns of genes under selection differed between the two genetic clusters.

## Discussion

### Genetic diversity and environmental determinants in *B. waltoni* populations

Heterozygosity is widely regarded as one of the key indicators of population genetic variation and is closely associated with the potential adaptive capacity of populations in response to environmental change [26, 27]. Based on whole-genome resequencing data, populations of *B. waltoni* in the northeastern Qinghai–Tibetan Plateau showed relatively similar levels of genetic diversity overall. The consistent excess of observed over expected heterozygosity and negative *F*is values suggest an absence of recent inbreeding and may reflect historical population connectivity or ongoing gene exchange among populations. Compared with reports on *B. terrestris* populations in Sweden [28], heterozygosity levels in *B. waltoni* were relatively higher, indicating that populations in the northeastern Qinghai–Tibetan Plateau retain appreciable genetic variation. In contrast, diversity metrics were lower than those reported for *Apis cerana* in Guizhou [29] and *B. morio* in southern Brazil [30]. Such interspecific differences likely reflect variation in demographic history, habitat stability, and long-term environmental conditions experienced by different taxa.

Population genetic diversity is known to be influenced by both geographic variables and environmental factors. Geographic variables such as latitude and longitude may not directly determine genetic diversity, but they can reflect spatial gradients associated with dispersal limitation, isolation by distance, historical colonization processes, and unmeasured environmental heterogeneity [31, 32]. Environmental factors such as elevation, temperature, and precipitation may more directly influence population size, survival, reproduction, and local adaptation [33–35]. In this study, Pearson correlation analyses were used to explore the effects of geographic and environmental factors on the genetic diversity of *B. waltoni* populations. After reducing strong collinearity and applying multiple-testing correction, elevation was identified as the only environmental variable significantly associated with genetic diversity in *B. waltoni*. Both *uHe* and *π* showed highly significant positive correlations with elevation, indicating that populations from higher elevations harbored higher levels of genetic diversity. This pattern may be related to the ecological characteristics of *B. waltoni*. The subgenus *Mendacibombus*, to which *B. waltoni* belongs, is a typical high-altitude specialist lineage adapted to the cool climatic conditions of high-elevation environments [6]. In addition, based on our previous MaxEnt assessment of suitable habitats for different indicator bumblebee species on the Qinghai–Tibetan Plateau under current and future climate scenarios, future climate warming may shift the suitable distribution range of *B. waltoni* toward higher elevations [36]. Thus, high-elevation habitats may provide more suitable environmental conditions for this species, thereby helping to maintain larger effective population sizes and promoting the maintenance of higher levels of genetic diversity. In addition, although longitude and annual mean temperature were not significant after multiple-testing correction, the directions of correlation showed that both *uHe* and *π* were negatively associated with these two variables, which was generally consistent with their positive relationships with elevation. Within the study region, longitude, elevation, and temperature were not independent spatial gradients: areas with lower longitude tended to occur at higher elevations, and higher-elevation areas generally had lower annual mean temperatures. Therefore, these correlation trends provide supplementary support for the view that *B. waltoni* is better adapted to cool, high-elevation environments. Although the remaining environmental variables were not significant after multiple-testing correction, their correlation trends may still have biological relevance. Precipitation, temperature, and other environmental conditions often act together to influence insect survival, distribution, and population dynamics [37]. Accordingly, the spatial differences in genetic diversity among *B. waltoni* populations in the northeastern Qinghai–Tibetan Plateau may not be attributable to a single factor, but may instead reflect the combined effects of elevation, climate, habitat conditions, and population history.

### Population structure and gene flow in *B. waltoni*

By integrating the results of ADMIXTURE, PCA, and phylogenetic analyses, we identified a well-defined population genetic structure of *B. waltoni* across the study region. The ADMIXTURE analysis indicated that the 18 populations could be consistently divided into two major genetic clusters. Populations located in the northeastern part of the study area (GAC1, QIL1, and SHD1) formed a relatively independent genetic cluster, whereas the remaining populations from the southwestern region constituted the second cluster. This clustering pattern remained stable with increasing values of K, and the northeastern cluster maintained a distinct genetic composition, suggesting a relatively high degree of genetic independence. Notably, several southwestern populations that are geographically closer to the northeastern region, including MAD1, MAD2, MAQ1, TOD1, and GUN1, exhibited varying degrees of admixture in their ancestral components. In the PCA, two individuals from GUN1 also showed some separation from the main southwestern cluster along the PC2 axis. This may reflect a certain degree of genetic differentiation among southwestern populations or among individuals within the same population. In addition, the relatively limited sampling coverage around GUN1 may also have influenced this result. Together, these patterns suggest that populations near the boundary between the two genetic clusters may not have diverged in complete isolation from the northeastern cluster, but instead experienced genetic influence from adjacent genetic groups during their evolutionary differentiation. The high concordance among ADMIXTURE, PCA, and phylogenetic analyses further supports the robustness of the inferred population structure.

Genetic differentiation among populations was further evaluated using pairwise *F*st, a widely used metric for assessing population genetic divergence [38]. In this study, pairwise *F*st values ranged from 0.023 to 0.072, with an overall mean of 0.041. Specifically, *F*st values among northeastern populations ranged from 0.028 to 0.039 (mean = 0.033), while those among southwestern populations ranged from 0.023 to 0.042 (mean = 0.032). These results indicate that *B. waltoni* populations exhibit generally low levels of genetic differentiation, although moderate differentiation is evident between the northeastern and southwestern clusters. Consistent with these patterns, relatively high levels of gene flow were detected among populations, particularly between geographically proximate populations. The Mantel test further revealed a significant positive correlation between genetic differentiation and geographic distance, indicating a clear pattern of isolation by distance and suggesting that geographic distance partially constrains gene exchange among populations. AMOVA based on the ADMIXTURE-defined grouping (K = 2) showed that genetic variation among groups was significantly greater than variation among populations within groups, providing additional support for the presence of pronounced genetic structure between the northeastern and southwestern clusters.

Although population structure and genetic differentiation analyses revealed clear divergence between the northeastern and southwestern populations of *B. waltoni*, multiple lines of evidence indicate that this divergence did not occur under conditions of complete isolation. ABBA–BABA tests detected significant asymmetrical allele sharing in a subset of population trios, and F-branch analysis further revealed elevated *fb* values between several southwestern populations (e.g., MAD2, TOD1, and GUN1) and the northeastern cluster. These findings are consistent with the optimal TreeMix model (m = 4), which inferred directional historical gene flow from the northeastern lineage into multiple southwestern populations, including MAD2, MAD1, TOD1, and MAQ1. Importantly, populations exhibiting pronounced admixture signals in the ADMIXTURE analysis were the same populations that showed strong gene flow signals in the ABBA–BABA, F-branch, and TreeMix analyses. These populations are predominantly distributed in geographic transition zones between the northeastern and southwestern clusters, suggesting that they may have played an important role as conduits for gene exchange between distinct genetic lineages of *B. waltoni*. In contrast, no obvious signal of gene flow was detected within the northeastern populations. This may be related to the relatively high genetic similarity among these populations, which could make weaker internal gene exchange signals more difficult to detect. In addition, the relatively smaller number of sampled populations and individuals in the northeastern cluster may also have reduced the power to detect internal gene flow signals to some extent. A similar phylogeographic pattern has been reported in *B. pyrosoma*. Jiang et al. showed that populations in and around the Qinghai–Tibetan Plateau exhibited evidence of dispersal out of the plateau together with lineage divergence along elevational gradients, suggesting that the genetic structure of bumblebees in this region may be shaped by both dispersal and regional divergence, rather than by long-term isolation alone [39]. In this context, the strong historical gene flow detected between the northeastern populations of *B. waltoni* and several southwestern populations, particularly those distributed in geographic transition zones, suggests that transition-zone populations may have facilitated gene flow between partially differentiated lineages under the complex topography and environmental heterogeneity of the northeastern Qinghai–Tibetan Plateau.

### Selective sweep signals and functional enrichment

*F*st and θ*π* have been widely recognized as effective approaches for detecting genomic regions under selective sweeps, particularly when identifying functional loci associated with environmental adaptation [40]. Using a combined analysis of *F*st and log_2_(θπ_southwest_ / θπ_northeast_), we investigated selection signals across different population clusters of *B. waltoni*. Our results revealed pronounced differences in the functional enrichment patterns of genes under selection between the southwestern and northeastern clusters, providing evidence for potential differences in adaptive responses to contrasting environmental conditions.

In the southwestern cluster, which is distributed mainly across high-altitude regions of the Sanjiangyuan area, candidate genes under selection were significantly enriched in functional categories related to cellular structural reinforcement, hypoxia stress response, neural development, and metabolic remodeling centered on insulin signaling pathways. In high-altitude environments characterized by low oxygen availability and low temperatures, insects typically face constraints on respiration, energy metabolism, and enzymatic activity [41]. In this study, genes under selection in the southwestern cluster showed significant enrichment in functions such as calcium ion binding and regulatory ncRNA-mediated gene silencing. Calcium ions serve as critical intracellular second messengers and play key roles in cellular signal transduction under hypoxic conditions [42], contributing to the regulation of stress responses and metabolic homeostasis [43, 44]. The enrichment of post-transcriptional and non-coding RNA–mediated regulatory mechanisms further suggests that *B. waltoni* may rely on rapid and energy-efficient gene expression modulation to fine-tune protein synthesis in response to persistent and fluctuating high-altitude environmental stress [45]. KEGG enrichment analysis additionally revealed significant enrichment of genes involved in the insulin signaling pathway (Type II diabetes mellitus). This finding suggests that natural selection may have acted on insulin-related genes in the southwestern populations to optimize energy allocation and metabolic regulation, thereby enhancing survival and activity under extreme high-altitude conditions. Previous studies have shown that insects can induce tracheal system remodeling under hypoxic conditions through signaling molecules such as branchless, resulting in increased tracheal branching [46, 47]. Consistent with this, genes under selection in the southwestern cluster were significantly enriched in GO terms related to external encapsulating structures, extracellular matrix organization, cellular component organization, and microtubule regulation. These processes play fundamental roles in cytoskeletal reorganization and tissue structural regulation, potentially providing molecular support for hypoxia-induced tracheal remodeling. In addition to hypoxia and low temperature, bumblebees inhabiting high-altitude regions are chronically exposed to strong ultraviolet radiation and high solar irradiance. The enrichment of genes involved in cellular structural maintenance may therefore also contribute to maintaining cellular integrity and internal homeostasis under harsh environmental conditions. Neural regulation and behavioral adaptation are likewise critical for insects living in extreme environments [48]. In this study, candidate genes in the southwestern cluster were significantly enriched in neural development–related processes, including neuron differentiation and generation of neurons. These results suggest that natural selection may have targeted neural developmental programs to ensure the formation of a functional nervous system under hypoxic conditions, particularly for regulating respiratory rhythms, flight coordination, and cold adaptation—traits that are essential for individual survival and population persistence. Furthermore, the southwestern cluster is mainly distributed in the Sanjiangyuan region, where vegetation is relatively simple and dominated by alpine meadows [49]. Plant resources are limited at high elevations, and alpine plants often synthesize defensive compounds such as phenolics, flavonoids, and tannins to cope with extreme climatic conditions [50]. These exogenous compounds may be ingested by bumblebees during foraging and potentially affect their physiological performance [51, 52]. In this study, KEGG enrichment analysis identified significant enrichment of the caffeine metabolism pathway, although only a small number of genes were involved. This pathway is part of the cytochrome P450–mediated xenobiotic metabolism system [53]. Cytochrome P450 enzymes represent a core detoxification system in insects, enabling the oxidation, modification, and clearance of diverse plant secondary metabolites [54, 55]. The enrichment of this pathway suggests that the southwestern cluster of *B. waltoni* may have enhanced metabolic capacity and tolerance toward defensive plant compounds, facilitating more efficient utilization of limited floral resources to maintain energy supply and physiological stability in alpine environments.

In contrast to the southwestern cluster, the northeastern cluster of *B. waltoni* is distributed at higher latitudes but relatively lower elevations. Genes under selection in this cluster were significantly enriched in pathways related to developmental regulation, cellular structure formation, intracellular material transport, and the metabolism of plant-derived compounds. Previous studies have demonstrated that at higher latitudes, coordinated changes in photoperiod and temperature serve as critical cues regulating insect life-history traits [56]. As latitude increases, the growing season becomes shorter [57, 58], imposing strong temporal constraints on development and requiring insects to complete their life cycles within a limited time window [59]. In this study, candidate genes in the northeastern cluster were significantly enriched in core developmental processes such as dorsal/ventral pattern formation, as well as pathways closely associated with intracellular transport and signal regulation, including membrane trafficking and SNARE interactions in vesicular transport. These results suggest that northeastern populations may enhance developmental efficiency through fine-tuned regulation of developmental programs and associated cellular processes, thereby adapting to shorter growing seasons. Additionally, genes under selection in the northeastern cluster were enriched in functions related to the negative regulation of cell population proliferation and the regulation of small-molecule metabolic processes, indicating precise control over growth and metabolic rhythms. Such regulatory optimization may enhance adaptation to environments characterized by strong seasonality at higher latitudes. Although the northeastern cluster is distributed at higher latitudes, its relatively lower elevations provide more moderate environmental conditions that support plant growth and floral resource availability. In addition, previous studies have shown that plant richness is relatively high in eastern Qinghai [60], which may increase the diversity of plant secondary metabolites encountered by bumblebees during foraging. Consistent with this, KEGG enrichment analysis revealed significant enrichment of pathways such as terpenoid backbone biosynthesis and metabolism of terpenoids and polyketides, which are closely associated with the synthesis and metabolism of plant-derived secondary compounds. Terpenoids represent the largest and most diverse class of plant secondary metabolites [24] and serve as precursors for numerous insect pheromones and defensive compounds [61]. The enrichment of these pathways suggests that the northeastern cluster of *B. waltoni* may have evolved enhanced metabolic capacity to process diverse terpenoid compounds, facilitating adaptation to more complex and heterogeneous floral resource compositions.

Taken together, genes under selection exhibited markedly different functional enrichment patterns between the southwestern and northeastern clusters of *B. waltoni*, consistent with the contrasting altitudinal and latitudinal environmental gradients experienced by the two clusters. Selection in the southwestern cluster primarily reflects physiological adaptation to extreme high-altitude conditions, whereas in the northeastern cluster it is more closely associated with adaptation to strong seasonality and complex food resource environments. Although both clusters showed enrichment of pathways related to plant secondary metabolite metabolism, these pathways likely reflect enhanced detoxification capacity in alpine environments in the southwestern cluster and broader metabolic utilization of diverse plant compounds in the northeastern cluster.

## Conclusion

Based on whole-genome resequencing data, this study investigated the genetic characteristics and adaptive patterns of 139 *B. waltoni* individuals from 18 geographic populations in the northeastern Qinghai–Tibetan Plateau. The results indicate that *B. waltoni* populations showed relatively similar levels of genetic diversity, and that elevation was the only environmental factor significantly associated with genetic diversity after correction for multiple testing. Population genetic structure analyses revealed a clear division of *B. waltoni* populations into two major genetic clusters, corresponding to the northeastern and southwestern regions, with pronounced genetic structure but relatively low overall genetic differentiation. Although distinct genetic differentiation was detected, multiple lines of evidence from gene flow analyses suggest that this divergence did not arise under conditions of complete isolation. Importantly, differences in selective signals and functional enrichment patterns between the two genetic clusters reflect their contrasting adaptive strategies to high-altitude environments versus strong seasonality and complex floral resource conditions. Together, this study provides new genomic evidence for understanding the mechanisms of adaptive evolution in pollinating insects inhabiting high-altitude environments and offers a scientific basis for the conservation and management of key pollinator taxa in plateau ecosystems.

## Supplementary Information

Supplementary Material 1.

Supplementary Material 2.

## Data Availability

The datasets supporting the conclusions of this article are available in the Genome Sequence Archive (GSA) repository of the National Genomics Data Center [ https://ngdc.cncb.ac.cn/gsa], under accession number CRA039482. The datasets are currently under an embargo period set by the authors and will be publicly released at a later date.
