# Supplementary material for: Whole-genome resequencing reveals population genetic structure and selective sweeps in Bombus waltoni
Source: BMC Genomics. 2026 Jul 16;27:838. doi: 10.1186/s12864-026-13175-y (PMC13656426; doi:10.1186/s12864-026-13175-y)
Supplement: Supplementary file 1 [file 12864_2026_13175_MOESM2_ESM.docx]

Figure S1. Pairwise identity-by-descent relationships among 146 *B. waltoni* individuals

Figure S2. Pearson correlation matrices of geographic and environmental variables.
(a) Pairwise correlations among all candidate geographic and environmental variables.
(b) Pairwise correlations among the retained variables after excluding highly collinear predictors. Color intensity represents the magnitude of the correlation coefficient, with red indicating positive correlations and blue indicating negative correlations.

Figure S3. TreeMix analysis results showing inferred population graphs of *B. waltoni* under different migration scenarios. No outgroup was specified in the TreeMix analysis, and the resulting graphs are presented as unrooted trees.

Figure S4. OptM fit result map, which evaluated different m results of TreeMix, demonstrating the best fit of TreeMix for m = 4.
